# Supplementary material for: A study on the potential of higher education in reducing carbon intensity
Source: PLoS One. 2024 Nov 26;19(11):e0309546. doi: 10.1371/journal.pone.0309546 (PMC11594484; doi:10.1371/journal.pone.0309546)
Supplement: Attachment — Submitted filename: Response to reviewer 2s comments.pdf [file pone.0309546.s002.pdf]

Dear Editor and Reviewers:
Re: Manuscript ID: PONE-D-24-17034

We sincerely thank you and the reviewers for the very helpful comments and constructive suggestions that have led to significant improvements of our paper. We have carefully addressed your comments point-by-point in the revised manuscript.

The main changes and revisions include 1) We reworked the introduction section to further add the relationship between carbon emissions and higher education and to increase the marginal contribution of the article.; 2) We added 2.3 Theoretical analysis and research hypothesis to refine the research hypotheses; 3) In the empirical research section, we re-estimated and controlled for time fixed effects and province fixed effects, and reworked the heterogeneity analysis; 4) We reworked the abstract section to add quantitative results.

We hope that the changes and responses to comments in the revised manuscript are sufficient to make our manuscript suitable for publication in *PLOS ONE*.

Response to reviewer 2's comments

This article explores the relationship between carbon emissions and higher education, offering a unique and innovative perspective. However, several areas require improvement and further clarification:

Thank you very much for the reviewer's recognition of the topic and the following suggestions, we have carefully revised the article according to your suggestions, and the main changes are as follows: 1) We reworked the introduction section to further add the relationship between carbon emissions and higher education and to increase the marginal contribution of the article.; 2) We added Theoretical analysis and research hypothesis to refine the research hypotheses; 3) In the empirical research section, we re-estimated and controlled for time fixed effects and province fixed effects, and reworked the heterogeneity analysis; 4) We reworked the abstract section to add quantitative results.

1. The author examined the impact of higher education on carbon emissions. However, environmental awareness education at the primary or secondary school levels also affects carbon emissions. How did the author account for the potential interference from primary and secondary education on carbon emissions?

Response 1: 为了排除小学或中学阶段的环保意识教育也会影响碳强度，本文分别从高等教育投入与高等教育产出视角选择自变量，其中高等教育投入角度本文选取高等教育方面的人均公共预算支出作为各地区高等教育水平的指标;高等教育产出角度本文选取人均教育年限和高等教育的人口比例作为高等教育的代理变量，考虑到目前中国已普及义务教育，因此人均教育年限和高等教育人口比例可以很好的反应中国高等教育产出情况。In order to rule out the influence of environmental awareness education at the elementary and secondary school levels on carbon intensity, this paper selects independent variables from the perspectives of higher education inputs and outputs. For the perspective of higher education inputs, this paper chooses the per capita public budget expenditure in higher education as an indicator of the level of higher education in various regions. From the perspective of higher education outputs, this paper selects the average years of education per capita and the proportion of the population with higher education as proxy variables for higher education. Considering that compulsory education has been universalized in China, the average years of education per capita and the proportion of the population with higher education can effectively reflect the outputs of higher education in China.

This approach allows for a more effective demonstration of the variations in investment in higher education by local governments. The specific measurement methodology adopted in this study is:

$$\mathrm{Edu} = \frac{\mathrm{Budget}}{\mathrm{Number}} \#(3)$$

In this analysis, 'Edu' denotes the level of local higher education, as reflected by the per capita public budget expenditure on higher education. 'Budget' signifies the public fiscal allocation for higher education, while 'Number' indicates the the number of students enrolled in local general colleges and universities.. Data pertaining to the public fiscal allocation for higher education and university enrollments are obtained from the China Education Expenditure Statistical Yearbook.

To verify the reliability of the conclusions and avoid incidental findings due to the selection of specific variables, this paper plans to change the core explanatory variable of higher education. Since higher education is defined from the perspective of input by the per capita general public

budget expenditure on education, This paper selects the average years of education per capita and the proportion of the population with higher education as proxy variables for higher education from the perspective of higher education output. The calculation method for the average years of education per capita is shown in the formula, and the proportion of the population in higher education is represented by Formula 4; the proportion of the population in higher education is also expressed by Formula 5:

$$\mathrm{Eduyear} = \frac{\mathrm{Primary} * 6 + \mathrm{junior} * 9 + high * 12 + 16 * higher}{\mathrm{Number}} \#(4)$$

$$\mathrm{Eduratio} = \frac{higher}{\mathrm{Number}} \#(5)$$

Eduyear represents the average years of education per capita, primary denotes the number of individuals with primary school education, junior indicates the number of individuals with junior high school education, high stands for the number of individuals with high school education, higher signifies the number of individuals with education beyond college level, and number refers to the population aged six and above. The data are sourced from the China Statistical Yearbook and China Population Census data. The regression results, after substituting the higher education variable, are presented in Table 5.

2. In the Introduction section, the author explains the relationship between carbon emissions and higher education, but the discussion lacks depth. It is recommended that the author further elaborate on the relationship between higher education and carbon emissions by integrating existing research. Additionally, the author should consider reducing some of the common knowledge content in the Introduction.

Response 2: Thanks to the reviewers' suggestions, we have reconsolidated the introductory statements, reduced some of the attempted content, and clarified the marginal contributions of this paper（Lines 77-88）.

In addition, in order to further explain the relationship between carbon emissions and higher education, we added a section on Theoretical analysis and research hypothesis to more systematically analyze the theory and improve the research hypothesis（Lines 172-207）.

Lines 77-88： This study analyzes the relationship between higher education and carbon intensity across thirty provincial administrative regions in China using a fixed-effects model. To strengthen the robustness of our findings, we substituted the core explanatory variables, further validating our conclusions. The analysis then categorizes China into eastern and central-western regions to explore regional disparities in the impact of higher education on carbon intensity. Additionally, we developed a mediation effect model that elucidates the roles of technological innovation incentives, environmental awareness promotion, and industrial structure enhancement in mediating this relationship. Based on these insights, we offer targeted policy recommendations. In comparison to existing studies, this paper makes three primary contributions: First, it proposes a theoretical framework from the perspective of higher education, thereby broadening the environmental governance discourse. Second, it addresses both the spatial variability in higher education and its differential impacts on carbon intensity, considering both educational inputs and outputs. Third, it identifies critical mechanisms such as technological innovation, environmental awareness, and industrial upgrading that link higher education and carbon intensity.

Lines 172-207: **Theoretical analysis and research hypothesis**

From a behavioral economics standpoint, highly educated individuals typically exhibit a heightened sense of social responsibility. They are more attentive to environmental sustainability and actively oppose lifestyles and consumption habits characterized by high energy use. Such individuals are predisposed to adopting pro-environmental behaviors and demonstrating a robust environmental consciousness. In both professional and personal contexts, they prefer to efficiently utilize idle resources, opt for low-carbon transportation solutions, and use environmentally friendly products. For example, as consumers, educated individuals tend to favor purchasing electric vehicles to mitigate carbon emissions43. Furthermore, educated entrepreneurs and managers are more likely to uphold corporate social responsibilities, advocate for sustainable production methods, and minimize the ecological impact of their business operations.

According to human capital theory, education and training represent crucial investments in human capital. Higher education not only augments workers' professional knowledge and practical skills but also fosters their capacity for innovation and research. This comprehensive enhancement significantly improves their level of knowledge, professional abilities, and overall competencies, thereby increasing their potential future earnings and job prospects. The educational advancement of the workforce contributes to the accumulation and diversification of human capital, which, in turn, drives the growth of high-tech industries and the evolution of industrial structures. Consequently, this shift leads to the displacement of traditional high-pollution industries by more sustainable high-tech and green sectors, effectively reducing carbon intensity.

Theoretical economics suggests that higher education not only raises an individual's knowledge level but also generates knowledge spillover effects. This educational process not only benefits the learners themselves but also exerts a positive influence on the wider society. By engaging in both educational and research activities, universities can attract technology-driven enterprises to establish collaborative platforms such as joint laboratories, technology transfer centers, and innovation incubators. These platforms not only enable joint research projects and technological advancements but also facilitate the specialized training of innovative talent. They promote local technological innovation, catalyze the development of emerging clean technologies, improve resource efficiency in production, and ultimately contribute to a reduction in carbon intensity.

Based on the analysis above, this paper illustrates the discussed mechanisms in Figure 1 and proposes the following hypotheses:

Hypothesis 1: Higher education can significantly suppress carbon intensity.

Hypothesis 2: Higher education reduces carbon intensity through the mechanism of promoting environmental awareness.

Hypothesis 3: Higher education reduces carbon intensity through the mechanism of upgrading industrial structures.

Hypothesis 4: Higher education reduces carbon intensity through the mechanism of incentivizing technological innovation.

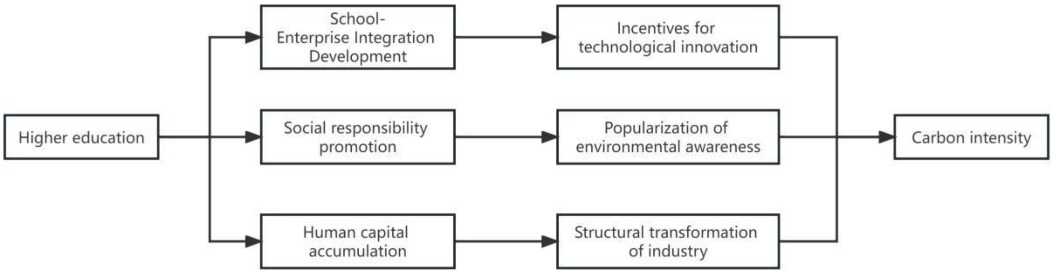

Fig 1. Mechanistic analysis of higher education and carbon intensity.

3. The author has obtained some quantitative conclusions, but these are not reflected in the abstract. It is recommended that the author include some quantitative conclusions in the abstract.

Response 3: Thanks to the reviewers' suggestions, we have reworked the abstract.

Abstract

The Chinese government has established definitive goals to reach a "carbon peak" by 2030 and achieve "carbon neutrality" by 2060. Investigating the attainment of these emission reduction objectives while simultaneously fostering regional economic growth and enhancing living standards holds critical importance. This study examines the link between higher education and carbon intensity across China's thirty provincial-level administrative regions, employing fixed effects models on provincial panel data spanning 2001-2020. The findings, validated through robustness tests and a mediation effect model, elucidate the mechanisms by which higher education influences carbon intensity. Notably, the results reveal that enhancing higher education markedly lowers carbon intensity; specifically, a 1% increase in the logarithmic transformation of per capita investment in higher education in a province decreases its carbon intensity by 0.219%. Additionally, higher education's output similarly contributes to reductions in carbon intensity. The influence of higher education on reducing carbon intensity is particularly pronounced in the central and western regions of China. Moreover, higher education facilitates the reduction of carbon intensity through mechanisms such as promoting environmental consciousness, advancing industrial structure, and encouraging technological innovation.
